# Development and Validation of Machine Learning Models to Classify Artery Stenosis for Automated Generating Ultrasound Report

**DOI:** 10.3390/diagnostics12123047

**Published:** 2022-12-05

**Authors:** Chih-Yang Yeh, Hsun-Hua Lee, Md. Mohaimenul Islam, Chiu-Hui Chien, Suleman Atique, Lung Chan, Ming-Chin Lin

**Affiliations:** 1 Graduate Institute of Biomedical Informatics, College of Medical Science and Technology, Taipei Medical University, Taipei 11031, Taiwan; 2 Department of Neurology, Taipei Medical University Hospital, Taipei Medical University, Taipei 11031, Taiwan; 3 Department of Neurology, School of Medicine, College of Medicine, Taipei Medical University, Taipei 11031, Taiwan; 4 Dizziness and Balance Disorder Center, Shuang Ho Hospital, Taipei Medical University, New Taipei City 23561, Taiwan; 5 Department of Neurology, Shuang Ho Hospital, Taipei Medical University, New Taipei City 23561, Taiwan; 6 International Center for Health Information Technology, College of Medical Science and Technology, Taipei Medical University, Taipei 11031, Taiwan; 7 Division of Operation Performance, Center for Management and Development, Taipei Medical University, Taipei 11031, Taiwan; 8 Department of Public Health Science, Faculty of Landscape and Society, Norwegian University of Life Sciences, 1430 Ås, Norway; 9 Department of Health Informatics, College of Public Health and Health Informatics, University of Hail, Hail 55476, Saudi Arabia; 10 Taipei Neuroscience Institute, Taipei Medical University, Taipei 11031, Taiwan; 11 Department of Neurosurgery, Shuang Ho Hospital, Taipei Medical University, New Taipei City 23561, Taiwan

**Keywords:** machine learning, artery stenosis, ultrasound report, transcranial doppler, extracranial carotid doppler

## Abstract

Duplex ultrasonography (DUS) is a safe, non-invasive, and affordable primary screening tool to identify the vascular risk factors of stroke. The overall process of DUS examination involves a series of complex processes, such as identifying blood vessels, capturing the images of blood vessels, measuring the velocity of blood flow, and then physicians, according to the above information, determining the severity of artery stenosis for generating final ultrasound reports. Generation of transcranial doppler (TCD) and extracranial carotid doppler (ECCD) ultrasound reports involves a lot of manual review processes, which is time-consuming and makes it easy to make errors. Accurate classification of the severity of artery stenosis can provide an early opportunity for decision-making regarding the treatment of artery stenosis. Therefore, machine learning models were developed and validated for classifying artery stenosis severity based on hemodynamic features. This study collected data from all available cases and controlled at one academic teaching hospital in Taiwan between 1 June 2020, and 30 June 2020, from a university teaching hospital and reviewed all patients’ medical records. Supervised machine learning models were developed to classify the severity of artery stenosis. The receiver operating characteristic curve, accuracy, sensitivity, specificity, and positive and negative predictive value were used for model performance evaluation. The performance of the random forest model was better compared to the logistic regression model. For ECCD reports, the accuracy of the random forest model to predict stenosis in various sites was between 0.85 and 1. For TCD reports, the overall accuracy of the random forest model to predict stenosis in various sites was between 0.67 and 0.86. The findings of our study suggest that a machine learning-based model accurately classifies artery stenosis, which indicates that the model has enormous potential to facilitate screening for artery stenosis.

## 1. Introduction

Stroke is the second leading cause of death globally, killing nearly 6.5 million people annually [1]. A transient ischemic attack (TIA), known as a “mini-stroke”, is a medical emergency caused by the temporary disruption of blood supply (i.e., ischemia) to the brain [2,3]. Previous studies reported that 20–23% of patients with ischemic stroke were associated with a TIA [4,5], and 80% of strokes after TIA are preventable [6]. Neurovascular specialists usually evaluate and determine existing carotid artery stenosis or occlusion (CAS) to manage the early phase of stroke and thus improve detection and therapy. Duplex ultrasonography (DUS) is a safe, non-invasive, and affordable primary screening tool to identify carotid artery stenosis. Although it has shown a high degree of sensitivity and specificity to TIA [7], the overall process of DUS examination is complex and produces a higher number of images to review [8,9].

Transcranial Doppler (TCD) and Extracranial Carotid Doppler (ECCD) are widely used imaging techniques in the acute phase of the stroke to detect the presence of stenosis. Several guidelines and diagnostic criteria are used to interpret and report a complete TCD and ECCD examination. These guidelines and criteria mentioned that DUS examination reports should be performed in grayscale images, spectral Doppler waveforms, spectral Doppler velocities, and color Doppler images [10,11,12]. The European Stroke Organization and the American Society of Neuroimaging Practice Guidelines recommended several high-velocity criteria for detecting, quantifying, and progressing CAS [13,14]. The lack of experienced clinicians, heavy workload, and time constraints are the major challenges to correctly identifying high-risk patients and obtaining the significant clinical advantage of using these techniques [15,16].

Over the past decade, machine learning models have shown excellent diagnostic performance in detecting potential medication conditions, including neurological diseases [17,18]. Machine learning-based clinical decision support systems (CDSS) may help to improve diagnostic performance for stenosis detection. Therefore, the main objective of our study was to develop a supervised machine learning model to automatically identify the critical factors of different neurovascular ultrasound examinations, whether artery stenosis exists or not.

## 2. Methods

### 2.1. Data Collection

**Figure 1:**
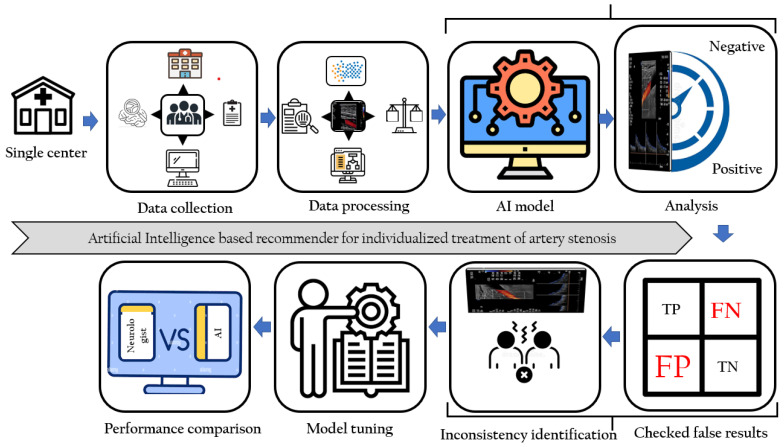
A schematic diagram of the study process.

The schematic diagram of our study is presented in Figure 1. The neurovascular ultrasound (TCD and ECCD) data were collected from 538 subjects retrospectively between 1 June 2020, and 30 June 2020, from a university tertiary hospital. Furthermore, the medical records of those patients were also reviewed to check their appropriateness. All of the neurovascular ultrasound examinations were performed by experienced registered vascular technologists and measured using a B-mode ultrasonography and color Doppler (Affiniti 50, Philips Ultrasound Inc., Bothell, WA, USA). Expert neurologists completed each ECCD and TCD examination order and report. Neurologists also independently reviewed their patient’s medical records with Doppler measurements (e.g., an estimate of blood flow), and ultrasonography images. These reports were evaluated based on their neurology knowledge and the multidisciplinary consensus criteria for the diagnosis of carotid and cerebral artery stenosis. The retrospective study was approved by the Taipei Medical University–Joint Institutional Review Board (approval No: N202011001).

### 2.2. Variables Collection

We collected the sonographic parameters from DICOM (Digital Imaging and Communications in Medicine) files and followed the standard protocol of neurosonology [10,19,20]. For the extracranial cerebrovascular—the common, internal, and external carotid arteries, and V2 segment of vertebral arteries (CCA, ICA, ECA, and VA, respectively) were examined using ECCD. Peak systolic velocity (PSV), end-diastolic velocity (EDV), blood flow volumes, and vascular diameters of the vessels were measured with pulsatility index (PI) or resistance index (RI) being calculated in all of the arteries. For the intracranial cerebrovascular—the anterior, middle, and posterior cerebral arteries, V4 segment of vertebral arteries, and the basilar arteries (ACA, MCA, PCA, VA, and BA, respectively) were also measured using TCD. The PSV, EDV and mean flow velocity (MFV) of the vessels were also measured.

### 2.3. Handling Imbalanced Dataset

As our dataset was imbalanced, we therefore used the Synthetic Minority Oversampling Technique (SMOTE), which allows synthetic samples to be generated for the minority category. The SMOTE approach was introduced by Nitesh Chawla in 2002 [21], which helps to balance the class distribution without providing any additional information to the model. This technique is used to overcome the overfitting problem posed by random oversampling.

### 2.4. Statistical Analysis

Statistical analyses were conducted by using PASW version 18.0 (SPSS Inc., Chicago, IL, USA). For categorical variables, we used descriptive statistics; however, the mean and standard deviation were calculated for continuous variables. Patient characteristics were compared between those who developed stenosis and those who did not, using Student’s *t*-tests and chi-square tests. *p* values less than 0.05 were considered statistically significant.

For model development, the dataset was randomly divided into a training set (80%) and a testing set (20%). We used 10-fold cross-validation and repeated it three times on the training set. This method involved dividing the training set into ten sets and using nine sets for training, and the remaining set was used for verification. This process was repeated ten times, and the results of the different test sets were averaged, ensuring an independent result from the actual dataset subdivision. In the training dataset, random forest (RF) and logistic regression (LR) were used to predict the development of stenosis using all the predictor variables. The accuracy, sensitivity, specificity, positive and negative predictive values, and the area under the receiver operating curve were used to measure the performance of the models. All analyses were performed using RStudio version 2022.2.2.485 for Windows (2009–2022 RStudio, PBC, Boston, MA, USA) and the RF package developed by Breiman and Culter in the R environment [22].

## 3. Results

### 3.1. Patient Selection

After retrospectively rereviewing neurovascular ultrasound examination reports, we observed several cases with inaccurate diagnoses by clinicians. We assessed medical errors and excluded those cases from our analysis where there was limited information (ECCD: 2.7%; TCD: 1.3%), miscalculation (ECCD: 1.0%), or incomplete report (ECCD: 0.4%). For example, the image of interpretation error showed that the PSVICA is more than 145 cm/s, but the ECCD report showed a normal flow Doppler signal of bilateral carotid arteries. Although those medical errors might not result in harm, they could lead to the wrong diagnosis of stroke. Finally, we selected 463 individuals with ECCD reports and 75 individuals with TCD reports. Detailed information regarding exclusion is presented in Table 1.

### 3.2. Patient Distribution

There were 463 subjects with ECCD reports (male: 235 (51%); female: 228 (49%). The percentage of right (Rt’) ICA and left (Lt’) ICA was similar in both groups; however, Rt’-VA was slightly higher in females than in males (100% vs. 97.9%). A higher number of female patients had VA total flow compared to male patients (100% vs. 96.6%). In the case of aberrant hemodynamics, Rt’-ICA, Lt’-ICA, Rt’-VA, and Lt’-VA were higher in male patients than in females. The characteristics of ECCD are shown in Table 2.

TCD reports were collected from 75 patients (male: 33 (44%); female: 42 (56%)). The mean age (sd.) of male and female patients was 69.7 (13.9) and 64.7 (13.3), respectively. Although male patients had high Rt’- and Lt’-MCA values, female patients had a high number of Rt’- and Lt’-VA values. The distribution of male and female patients for TCD is provided in Table 3.

### 3.3. Important Predictors of ECCD

We separated ECCD reports into right and left arteries (Rt’-ICA, Lt’-ICA, Rt’-VA, Lt’-VA) and total VA. The predictor variables of ICA were gender, age, PSV, and RI, while the predictor variables of VA were gender, age, diameter, RI, and blood flow rate. The predictor variables of total VA flow were gender, age, bilateral diameter, bilateral RI, and total blood flow rate. We calculated the most important predictor variables for RF using the Gini coefficient (Table 4). Table 4 shows that PSV was the explainable attribute for determining stenosis in unilateral ICA; RI and blood flow rate were the relatively explainable attributes for determining stenosis in unilateral VA, and the total blood flow rate was the explainable attribute for determining stenosis in bilateral VA.

### 3.4. Performance of Machine Learning Models to Predict Stenosis in ECCD

The performance of RF and LR is illustrated in Table 5. The RF model showed a better performance in terms of accuracy, sensitivity, and specificity than that of LR to classify stenosis. The accuracy, sensitivity and specificity for total VA was 1, 1, and 1, respectively, in the RF model.

**Figure 2:**
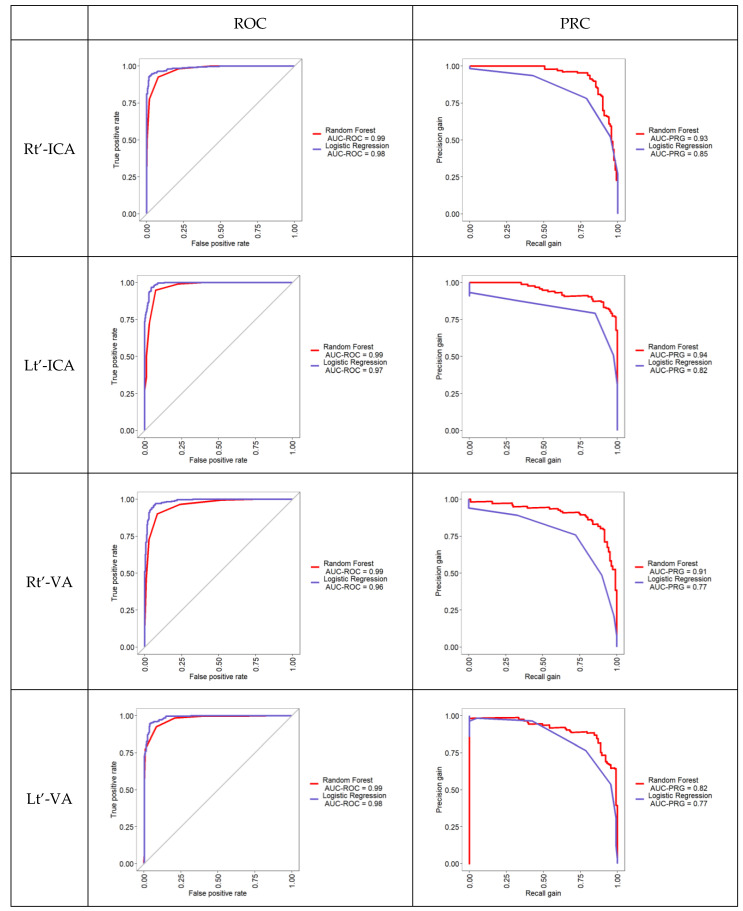
The area under the ROC curve and precision–recall curve for RF and LR model to classify artery stenosis in ECCD.

The overall area under the receiver operating curve of RF model for classifying artery stenosis in various sites was between 0.99 and 1. The range of the precision–recall curve for RF was between 0.82 and 0.93. The overall area under the receiver operating curve of LR model for classifying artery stenosis in different sites was between 0.96 and 1. The range of the precision–recall curve for LR was between 0.77 and 0.99 (Figure 2).

### 3.5. Important Predictors of TCD

The TCD report was also separated into Rt’-MCA, Lt’-MCA, Rt’-VA, Lt’-VA, and BA. The predictor variables of MCA were gender, age, M1 Dist./Prox. PSV, M1 Dist./Prox. PI, M2 PSV, and M2 PI. The predictor variables of VA or BA were gender, age, PSV, and PI (Table 6). Table 6 shows that M1 PSV was the explainable attribute for stenosis in unilateral MCA; PI was the explainable attribute for stenosis in unilateral VA, and PI was the explainable attribute for stenosis in the BA determinant.

### 3.6. Performance of AI Models to Predict Stenosis in TCD

The overall performance of RF and LR is presented in Table 7. The accuracy of Rt’-MCA, Lt’-MCA, Rt’-VA, Lt’-VA, and BA was 0.86, 0.67, 0.60, 0.73, and 0.80, respectively, in the RF. The RF model showed high sensitivity and specificity for predicting stenosis in Rt’-MCA and Lt’-MCA.

**Figure 3:**
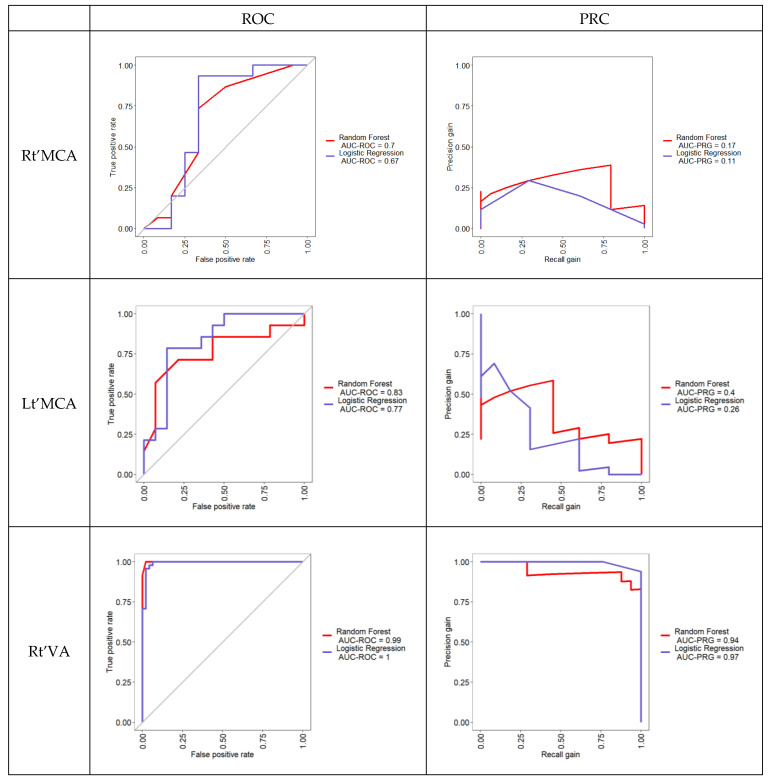

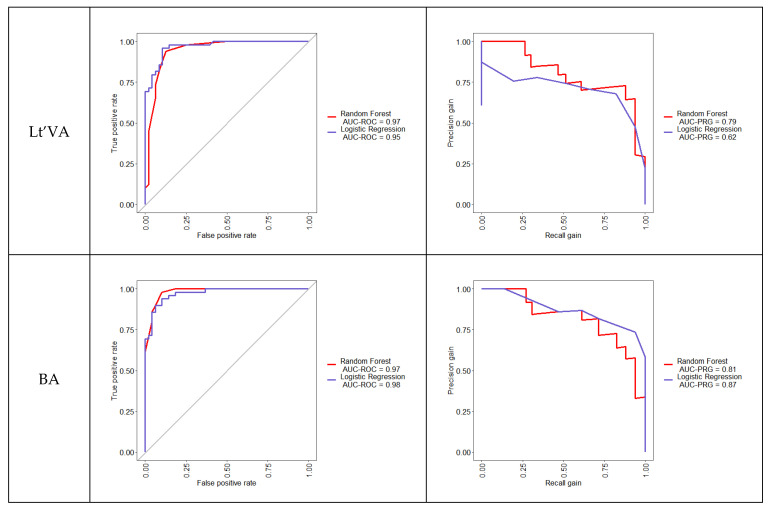
The area under the ROC curve and precision–recall curve for RF and LR to classify artery stenosis in TCD.

The overall area under the receiver operating curve of the RF model for classifying artery stenosis in various sites was between 0.70 and 0.99. The range of the precision–recall curve for RF was between 0.17 and 0.94. The overall area under the receiver operating curve of the LR model for classifying artery stenosis in different sites was between 0.67 and 1.00. The range of the precision–recall curve for LR was between 0.11 and 0.97 (Figure 3).

### 3.7. Manual Evaluation of Inconsistency

We used the confusion matrix of our RF model to retrieve the inconsistent cases which were incorrectly classified as positive or negative in the test dataset. A neurologist was invited to review original reports with hemodynamic information and compare the reports for appropriateness.

**Figure 4:**
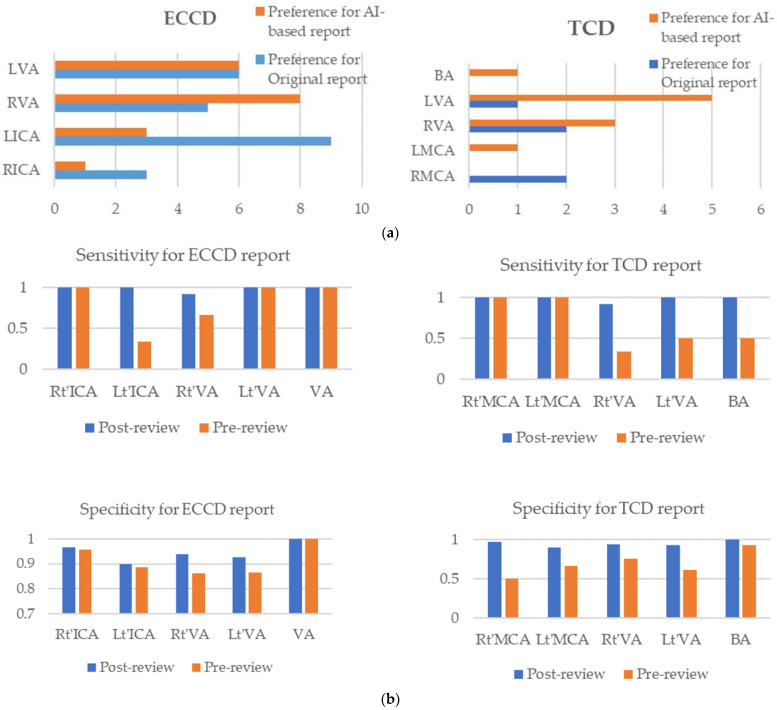
(**a**) The preference of manual and machine learning-based report for classifying artery stenosis. (**b**) The performance of random forest model after reviewing false positive and negative cases.

After reviewing the original ECCD reports with hemodynamic information, the neurologist considered that the original report was appropriate for ICA and the machine learning model generated reports were more appropriate for VA cases (Figure 4). When the neurologist reviewed the original TCD reports, he considered machine learning-based reports more appropriate than the original TCD reports, except for Rt’-MCA cases. Detailed information is provided in Table 8.

Finally, we recalculated the evaluation metrics of the RF model, including accuracy, sensitivity, and specificity. The accuracy of the RF model was significantly increased after reviewing the inconsistent cases. For ECCD, the overall sensitivity of the RF model was close to 1 for all sites. However, the overall specificity of TCD for classifying artery stenosis of all sites was also close to 1.

## 4. Discussion

Neurovascular ultrasound is a standard, painless, non-invasive imaging technique in the diagnosis of carotid and vertebral artery diseases [22,23]. This examination is performed by an experienced neurosonologist, who provides comprehensive and reliable information on the morphological and hemodynamic status of artery diseases. Manual reviewing of ECCD and TCD reports often leads to misclassification, wasteful duplication, and delayed diagnosis [24]. Machine learning-based CDSS can improve the review process, reduce misclassification, and enhance patient safety and care quality; therefore, machine learning-based CDSS was developed to accurately classify artery stenosis. The RF model performed excellently both in ECCD, and TCD reports in terms of sensitivity and specificity. For ECCD reports, the area under the receiver operating characteristics curve (AUC-ROC) and the precision–recall curve (AUC-PRC) was 0.99 and 0.93, respectively, in ICA. The AUC-ROC and AUC-PRC were 1.00 and 0.94, respectively, in total VA flow. For the TCD report, the RF model achieved an AUC-ROC of 0.98 and an AUC-PRC of 0.87 in BA.

In our study, the RF model identified several potential predictors to accurately classify artery stenosis. PSV was a key variable to determine stenosis in unilateral ICA by RF; however, we also validated it through manual review. AIUM Practice Parameter Ultrasound Examination of the Extracranial Cerebrovascular System [12] suggested that the areas of stenosis or suspected stenosis must be adequate to determine the maximal peak systolic velocity associated with the stenosis. Several potential velocity errors related to incorrect angle assignment increase with the Doppler angle. If stenosis above 50% in ICA should be graded to within a range, it would provide adequate information for clinical decision making. Kang et al. [25] reported that among all ultrasonographic features, only a higher PSV/EDV ratio helped to detect and was positively associated with an increased risk of ischemic stroke. Murry et al. [10] also reported that PSV is used to determine stenosis, and higher PSV is observed when contralateral ICA occlusion is present. However, the accuracy of examination results may vary due to operator expertise. To support the previous reports, Kim et al. [26] demonstrated that PSV may be a strong identifier of the degree of stenosis, especially in cases of ≥70% stenosis.

Moreover, RI and blood flow rate were potential variables to identify stenosis in unilateral VA. Previous studies mentioned that higher RI and lower blood flow velocity of carotid arteries is associated with ischemic stroke [27,28]. The duplex ultrasonographic examination of the extracranial arteries identify that vertebral artery blood flow significantly contribute to a “hemodynamic effect” of carotid disease [29]. The relationship between systemic arterial stiffness and cerebral circulation parameters is inconclusive; therefore, Kwater et al. [30] evaluated the association between pulsatility (PI) and RI of the MCA, reporting that increased PI and RI of MCA were potential contributors to increased aortic stiffness. PI of the MCA incorporated with PSV/EDV is associated with ischemic stroke [25].

### Limitation

Our study has several limitations. First, we collected only one-month ECCD and TCD reports to develop and test our model’s performance. However, those included all the doctors’ assessment varieties. The inclusion of more reports would help to develop a more robust model. Second, we collected data from one hospital; therefore, the included patients’ demographic characteristics might be the same and model performance may vary if we implemented it in other hospitals or countries. Third, identifying the severity, location, extent, and possible etiology of abnormality requires more information, including waveform [31]; however, only hemodynamic information (measurement of blood velocities) was used. Although our current model showed excellent performance for identifying stenosis using those simple variables, there may be a bias in stratifying carotid artery disease by duplex ultrasound. The accurate measurement of blood velocities can be used together with a qualitative assessment of the appearance of the stenosis, including the residual lumen diameter when visualized.

## 5. Conclusions

The findings of our study demonstrate that machine learning models, especially the random forest model, can accurately classify patients with artery stenosis using hemodynamics information. Compared with the manual classification of stenosis, the classification performance of the RF model was superior to most but not all parts, such as Lt’-ICA and Rt’-ICA. While RF is a simple and explainable model, its implementation on electronic health record systems can be deployed. Our model could assist all physicians with different training and experience in accurately classifying stenosis.

## Figures and Tables

**Table 1:** The key reason for exclusion.

Item	Root Cause	ECCD	TCD	Total
1	Incomplete report	2	0	2
2	Misplacement of category	13	1	14
3	Detect values are different with report comments	5	0	5
Total		20	1	21

**Table 2:** Patient distribution for ECCD.

Characteristics	Female (n = 228)	Male (n = 235)
Age (mean ± SD, range, yrs.)	63.0 ± 14.5 (18–96)	63.9 ± 14.1 (19–99)
Site of evaluation (no. of patients (%))		
Rt’-ICA	227 (99.6)	232 (98.7)
Lt’-ICA	227 (99.6)	232 (98.7)
Rt’-VA	228 (100.0)	230 (97.9)
Lt’-VA	227 (99.6)	231 (98.3)
VA Total Flow	228 (100.0)	227 (96.6)
Aberrant hemodynamics (no. of patients (%))		
Rt’-ICA	3 (1.3)	10 (4.3)
Lt’-ICA	2 (0.9)	13 (5.5)
Rt’-VA	10 (4.4)	22 (9.4)
Lt’-VA	9 (3.9)	12 (5.1)
VA Total Flow	26 (11.4)	36 (15.3)

**Table 3:** Patient characteristics for TCD.

Characteristics	Female (n = 42)	Male (n = 33)
Age (mean ± SD, range, yr.)	64.7 ± 13.3 (39–96)	69.7 ± 13.9 (39–95)
Site of evaluation (no. of patients (%))		
Rt’-MCA	12 (28.6)	22 (66.7)
Lt’-MCA	10 (23.8)	22 (66.7)
Rt’-VA	42 (100.0)	33 (100.0)
Lt’-VA	42 (100.0)	33 (100.0)
BA	42 (100.0)	32 (97.0)
Aberrant hemodynamics (no. of patients (%))		
Rt’-MCA	4 (33.3)	12 (54.5)
Lt’-MCA	3 (30.0)	12 (54.5)
Rt’-VA	7 (16.7)	8 (24.2)
Lt’-VA	6 (14.3)	3 (9.1)
BA	5 (11.9)	4 (12.5)

**Table 4:** Important predictor variables for RF model.

Random Forest	Predictors	Mean Decrease Gini
Rt’-ICA	Gender	13.44
	Age	80.74
	PSV	77.85
	RI	49.57
Lt’-ICA	Gender	3.12
	Age	61.84
	PSV	86.56
	RI	69.23
Rt’-VA	Gender	1.44
	Age	35.16
	Diameter	30.17
	RI	60.02
	Flow rate	75.95
Lt’-VA	Gender	1.15
	Age	30.28
	Diameter	30.04
	RI	89.89
	Flow rate	60.92
Total VA	Gender	0.55
	Age	8.21
	Rt’-diameter	18.95
	Rt’-RI	7.52
	Lt’-diameter	12.30
	Lt’-RI	10.53
	Total Flow rate	114.22

**Table 5:** The performance of the machine learning models to predict artery stenosis in various sites.

Specific Side/Artery	Model Performance	Accuracy	Sensitivity	Specificity	PPV	NPV
Rt’-ICA	RF	0.96	1.00	0.96	0.33	1.00
	LR	0.87	1.00	0.87	0.14	1.00
Lt’-ICA	RF	0.87	0.33	0.89	0.09	0.98
	LR	0.89	0.33	0.91	0.11	0.98
Rt’-VA	RF	0.85	0.67	0.86	0.25	0.97
	LR	0.82	0.67	0.83	0.21	0.97
Lt’-VA	RF	0.88	1.00	0.88	0.27	1.00
	LR	0.85	1.00	0.84	0.22	1.00
Total VA	RF	1.00	1.00	1.00	1.00	1.00
	LR	0.99	0.92	1.00	1.00	0.99

PPV: Positive Predictive Value; NPV: Negative Predictive Value.

**Table 6:** Key predictors of TCD in the RF model.

Random Forest	Predictors	Mean Decrease Gini
Rt’-MCA	Gender	0.18
	Age	0.94
	Dist. M1 PSV	1.13
	Dist. M1 PI	1.48
	Prox. M1 PSV	1.55
	Prox. M1 PI	1.20
	M2 PSV	1.19
	M2 PI	1.03
Lt’-MCA	Gender	0.17
	Age	0.77
	Dist. M1 PSV	2.18
	Dist. M1 PI	0.77
	Prox. M1 PSV	1.06
	Prox. M1 PI	1.40
	M2 PSV	1.16
	M2 PI	1.29
Rt’-VA	Gender	0.21
	Age	3.00
	PSV	8.41
	PI	18.69
Lt’-VA	Gender	0.44
	Age	8.35
	PSV	6.21
	PI	15.82
BA	Gender	0.47
	Age	6.07
	PSV	6.27
	PI	17.99

**Table 7:** Performance of RF and LR model to predict stenosis in various sites.

Specific Side/Artery	Model Performance	Accuracy	Sensitivity	Specificity	PPV	NPV
Rt’-MCA	RF	0.86	1.00	0.75	0.75	1.00
	LR	0.71	1.00	0.50	0.60	1.00
Lt’-MCA	RF	0.67	1.00	0.33	0.60	1.00
	LR	0.67	1.00	0.33	0.60	1.00
Rt’-VA	RF	0.60	0.33	0.67	0.20	0.80
	LR	0.60	0.33	0.67	0.20	0.80
Lt’-VA	RF	0.73	0.50	0.77	0.25	0.91
	LR	0.53	0.50	0.54	0.14	0.88
BA	RF	0.80	0.50	0.85	0.33	0.92
	LR	0.87	1.00	0.85	0.50	1.00

**Table 8:** Performance evaluation between the original and AI-based reports by expert neurologist.

Exam.	Specific Side/Artery	Inconsistent Cases	# of Preference for Original Report	# of Preference for Machine Learning-Based Report
ECCD	Rt’-ICA	4	3	1
	Lt’-ICA	12	9	3
	Rt’-VA	13	5	8
	Lt’-VA	12	6	6
	Total VA	0	0	0
TCD	Rt’-MCA	2	2	0
	Lt’-MCA	1	0	1
	Rt’-VA	5	2	3
	Lt’-VA	6	1	5
	BA	1	0	1

## Data Availability

Not applicable.
